# Exploring 3D Human Action Recognition Using STACOG on Multi-View Depth Motion Maps Sequences

**DOI:** 10.3390/s21113642

**Published:** 2021-05-24

**Authors:** Mohammad Farhad Bulbul, Sadiya Tabussum, Hazrat Ali, Wenli Zheng, Mi Young Lee, Amin Ullah

**Affiliations:** 1 Department of Mathematics, Jashore University of Science and Technology, Jashore 7408, Bangladesh; farhad@just.edu.bd (M.F.B.); sadiya.just.bd@gmail.com (S.T.); 2 Department of Electrical and Computer Engineering, Abbottabad Campus, COMSATS University Islamabad, Abbottabad 22060, Pakistan; hazratali@cuiatd.edu.pk; 3 School of Science, Xi’an Shiyou University, Xi’an 710065, China; wlzheng@xsyu.edu.cn; 4 Intelligent Media Laboratory, Department of Software, Sejong University, Seoul 143-747, Korea; 5 CORIS Institute, Oregon State University, Corvallis, OR 97331, USA

**Keywords:** 3D action recognition, depth motion maps, 3D auto-correlation features, decision fusion, Regularized Collaborative Representation Classifier (CRC)

## Abstract

This paper proposes an action recognition framework for depth map sequences using the 3D Space-Time Auto-Correlation of Gradients (STACOG) algorithm. First, each depth map sequence is split into two sets of sub-sequences of two different frame lengths individually. Second, a number of Depth Motion Maps (DMMs) sequences from every set are generated and are fed into STACOG to find an auto-correlation feature vector. For two distinct sets of sub-sequences, two auto-correlation feature vectors are obtained and applied gradually to $L_{2}$-regularized Collaborative Representation Classifier ($L_{2}$-CRC) for computing a pair of sets of residual values. Next, the Logarithmic Opinion Pool (LOGP) rule is used to combine the two different outcomes of $L_{2}$-CRC and to allocate an action label of the depth map sequence. Finally, our proposed framework is evaluated on three benchmark datasets named MSR-action 3D dataset, DHA dataset, and UTD-MHAD dataset. We compare the experimental results of our proposed framework with state-of-the-art approaches to prove the effectiveness of the proposed framework. The computational efficiency of the framework is also analyzed for all the datasets to check whether it is suitable for real-time operation or not.

## 1. Introduction

**Figure 1:**
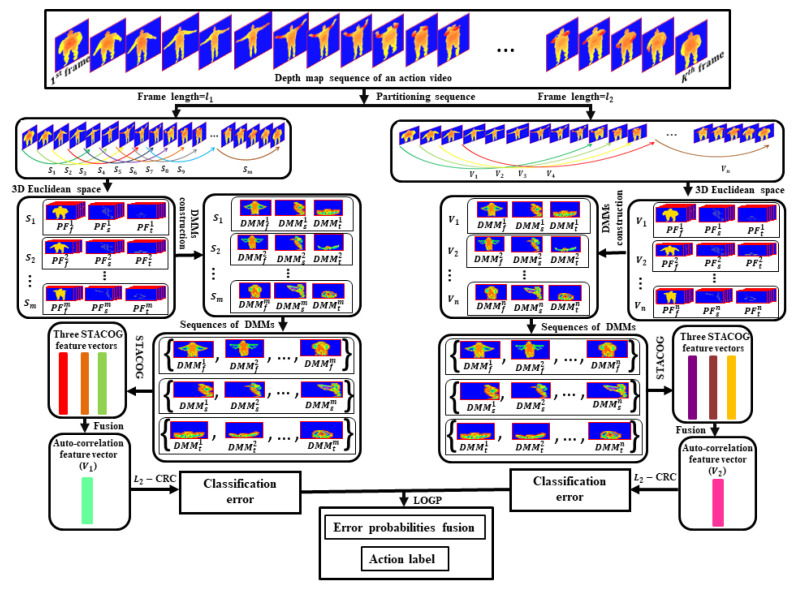
Proposed action recognition framework.

Human action recognition is one of the most challenging tasks in the area of artificial intelligence and has obtained attention due to widespread real-life applications, which extend from robotics to human-computer interface, automated surveillance system, healthcare monitoring, etc. [1,2,3]. Human actions are composed of contemporary behaviors of human body parts. The objective of human action recognition is to recognize actions automatically from an unlabeled video [4,5]. To capture human actions, there are two broad categories of devices based on wearable sensors and video sensors. In the prior, using these apparatuses many research works have been completed in the area of action recognition. To recognize wearable sensor-based actions, multiple sensors are connected to the human body. To obtain action information, most of the researchers have used different sensors such as accelerometers, gyroscopes, and magnetometers [6,7,8]. These wearable sensors are used in the healthcare system, worker monitoring, interactive gaming, sports, etc. However, they are not acceptable in all the domains of action recognition, for example in the automatic surveillance system. It is far from convenient for humans (especially patients) to wear the sensors for a long time and relatively it is difficult in cases of energy costs. Wearable sensors can have health risks. For those carrying smartphones, laptops, and tablets, wearable sensor increases exposure to radio wave. Although the use of multiple sensors increases recognition accuracy, it has limitations for real-life applications because of increased associated complexity and the cost of the total procedure. Because of the difficulties of wearable sensors, video sensors such as RGB cameras are used to recognize the action. RGB images give restricted 2D data as grayscale or RGB intensity rate, motion illegibility (e.g., color and texture variations), inflexibility in the foreground or background segmentation, illumination variation, and low resolution which resist recognizing action accurately [9,10]. With the emergence of advanced technology, the redemption of accessible depth sensors is broadly used to achieve 3D action information. 3D information can be obtained through three approaches. The first approach is costly marker-based motion capture systems (MoCap) which uses visual sensing of markers settled in different parts of the human body and triangulation from several cameras to gain three-dimensional spatial information and the human skeleton. In the second approach, a stereo camera is used to acquire 3D depth information [11]. The stereo camera consists of two or more lenses with an individual image sensor or film frame for each lens. A stereo camera gives depth information by stereo matching and distance computation from lenses to object. The images captured by a stereo camera are sensitive to light changes and background clutter and action recognition from such images is a very challenging task [12]. The third approach involves the use of a depth sensor (for example Microsoft Kinect) that gives real-time 3D information for human body parts [11]. Unlike RGB camera, depth sensor camera gives overlapping multiple body portion information, it is insensitive to light changes that improve performance at dark, and in such data, it is easy to normalize the body orientation or its size variations [9]. This camera gives depth information from which skeleton data is obtained. The studies based on skeletal data often show high recognition performance, but where skeletal data is not available, the studies are not robust in terms of accuracy. These discussions encourage us to use depth information to establish an action recognition framework. However, DMMs based on the total depth frames of the entire video are not capable of obtaining the total motion information. To reduce this disability, in this paper, the depth map sequence of the entire video are partitioned into a set of overlapping portions. Each portion contains the same number of depth frames and DMMs sequences are constructed from DMMs of all portions. Then, the entire depth video is described through 3D auto-correlation features obtained from DMMs sequences. With the calculated features, the $L_{2}$-regularized Collaborative Representation Classifier ($L_{2}$-CRC) [13] and the Logarithmic Opinion Pool (LOGP) rule [14] work jointly to assign an action label of the video. The proposed framework is visualized in Figure 1.

**Motivation and Contributions:**

The method proposed by Chen et al. [15], used 3D auto-correlation features from depth map sequences for action recognition; however, their framework has limited performance with the same data. They did not achieve significant results through their framework. Therefore, the objective of our work is to develop a framework to increase the recognition results as well as the overall performance by using the 3D auto-correlation gradient features.

The main contributions of our work are listed below:

- The depth map sequences of each action video are partitioned into a set of sub-sequences of equal size. Afterward, DMMs are created from each sub-sequence corresponding to three projection views (front, side, and top) of 3D Euclidean space. Then, three DMMs sequences are derived by organizing all the DMMs along the projection views. The video is fragmented by two times generating two sets of sub-sequences using two different frame lengths and thus there are two sets of three DMMs sequences are obtained.
- Our recognition framework mines the 3D auto-correlation gradient feature vectors from three DMMs sequences by using the STACOG feature extractor instead of mining from depth map sequences as shown in [15].
- A decision fusion scheme is applied to combine residual outcomes obtained for two 3D action representation vectors.
- The proposed framework achieved the highest results as compared to all the other work done by applying the STACOG descriptor on depth video.

The remainder of this paper is organized as follows. A couple of action recognition frameworks are reviewed in Section 2. The proposed framework is described in Section 3. In Section 4, experimental results and discussion of the proposed framework are reported. Finally, the conclusion and future research directions are presented in Section 5.

## 2. Related Work

This section describes current depth maps-based action recognition frameworks. Additionally, it also reviews skeleton, RGB, inertial, and fusion-based frameworks. Depending on depth data, Chen et al. [16] used local binary patterns (LBPs) to extract features. They represented two types of fusion levels and used the Kernel-based Extreme Learning Machine (KELM) for both levels. Ref. [17] introduced DMM-CT-HOG feature extractor that depends on Depth Motion Maps (DMMs), Contourlet Transform (CT), and Histogram of Oriented Gradients (HOGs). To improve accuracy, [18] used texture and dense shape information and combined them into DLE features that are fed to $L_{2}$-regularized Collaborative Representation Classifier ($L_{2}$-CRC). Ref. [19] proposed a method that fused classification results obtained by using multiple classifiers Kernel-based Extreme Learning Machine (KELM) through three types of features. A Bag-of-Map-Words (BoMW) method is introduced in [20] and feature vectors are extracted from Salient Depth Map (SDM) and Binary Shape Map (BSM) respectively and combined by the BoMW. Ref. [21] submitted a method using gradient local auto-correlations (GLAC) feature description algorithm based on spatial and orientational auto-correlations of local image. They introduced a fusion method depend on the Extreme Learning Machine classifier (ELM). Ji et al. [1], proposed a Spatio-Temporal Cuboid Pyramid (STCP) which subdivides the Depth Motion Sequence into spatial cuboids and temporal segments and used Histograms of Oriented Gradients (HOG) features. Chen et al. [22], used the texture feature descriptor Local Binary Pattern (LBP) and used the Kernel-based Extreme Learning Machine (KELM) classifier [19] to detect action. Again, in [23], DMMs are used as the feature descriptor. In their method, classification is accomplished by $L_{2}$-CRC consisting of a distance-weighted Tikhonov matrix. A new feature named Global Ternary Image (GTI) was introduced in [24]. By a bag of GTI model, the authors in [24] obtained data from motion regions and motion directions. After that, Liang et al. [25], used multiscale HOG descriptors and extracted local STACOG features. Then actions were recognized by $L_{2}$-CRC classifier. To improve accuracy, [15] fused 2D and 3D auto-correlation of gradients features which are extracted by Gradient Local Auto-Correlations (GLAC) and STACOG descriptors, respectively. Then, the action is classified by KELM with RBF kernel. Liu et al. [26] presented a method that used Adaptive Hierarchical Depth Motion Maps (AH-DMMs) and Gabor filter. Their method can extract motion and shape cues without decreasing temporal information and adopt the Gabor filter to encode the texture data of AH-DMMs. Jin et al. [27] split depth maps into a set of sub-sequences to create a vague boundary sequence (VB-sequence). They obtained dynamic features by combining all DMMs of VB-sequences. After that, Zhang et al. [28], presented low-cost 3D histograms of texture feature descriptors by which discriminant features are obtained. They also introduced a multi-class boosting classifier (MBC) to use different features for recognition. Furthermore, Chen et al. [29] introduced a multi-temporal DMMs descriptor in which a non-linear weighting function is used to assemble depth frames. They used a patch-based Local Binary Pattern (LBP) feature descriptor to obtain texture information. They used Fisher kernel representation and used the KELM classifier [19] for action classification. Li et al. [30], extracted texture features by discriminative completed LBP (disCLBP) descriptor and used a hybrid classifier associated with Extreme Learning Machine (ELM) and collaborative representation classifier (CRC). The authors in [31] used Histogram of Oriented Gradients (HOG) and Pyramid Histogram of Oriented Gradients (PHOG) as shape feature descriptors. They used $L_{2}$-CRC classifier. Azad et al. [32], introduced a multilevel temporal sampling (MTS) scheme that depended on the motion energy of depth maps. They extracted histograms of gradient and local binary patterns from a weighted depth motion map (WDMM). In [33], an action recognition scheme based on two types of depth images (generated using 3D Motion Trail Model (3DMTM)) was introduced. They obtained two features by using the GLAC algorithm from the images respectively and the features were fused in a vector. In the same year, Weiyao et al. [34] submitted Multilevel Frame Select Sampling (MFSS) model to obtain temporal samples from depth maps. They also proposed motion and static maps (MSM) and extracted texture features by the block-based LBP feature extraction scheme. They used the fisher kernel representation method to fuse obtained features and the KLM classifier to detect action. After that, Shekar et al. [35] introduced Stridden DMMs from which effective information of actions can be obtained quickly. They Undecimated the Dual-Tree Complex Wavelet Transform algorithm to extract wavelet (UDTCWT) features from the proposed DMMs. They used a Sequential Extreme Learning Machine classifier. To improve results, [36] used two types of images that are obtained by using the 3D Motion Trail Model (3DMTM). In their method feature vectors are mined from MHIs and SHIs by the GLAC feature descriptor. Al-Faris et al. [37] presented the construction of a multi-view region-adaptive multi-resolution-in-time depth motion map (MV-RAMDMM). They trained several scenes and time resolutions of the region-adaptive depth motion maps (RA-DMMs) by multi-stream 3D convolutional neural networks (CNNs). They used a multi-class SVMs classifier to recognize human actions.

Additionally, in [38], depth and inertial sensor-based features were extracted and fused to a single feature. The final feature set was passed to the collaborative representation classifier. Based on skeleton information, Youssef et al. [39], extracted normalized angles of local joints and used modified spherical harmonics (MSHs) to model the angular skeleton. They used MSH coefficients of the joints as the discriminative descriptor of the depth maps. Hou et al. [40], proposed a framework to convert Spatio-temporal data from skeleton sequence into color texture images. They used convolutional neural networks to obtain discriminative features. The authors in [41] created a Deep Convolutional Neural Network (3D2CNN) to acquire Spatio-temporal features from depth maps and calculated *JointVectors* from depth maps. The spatio-temporal features and JointVectors were passed individually to the SVM classifier and the outputs were combined into a single result. To improve accuracy [42] introduced a Spatially Structured Dynamic Depth Images $S^{2}$ DDI to represent an action video. To generate $S^{2}$ DDI, they presented a non-scaling method and approved a multiply score fusion scheme to increase accuracy. Using RGB image, Al-Obaidi et al. [43] presented a method to anonymize action video. Histograms of oriented gradients (HOG) features are extracted from anonymized video images. A Generative Multi-View Action Recognition (GMVAR) method is presented in [44], by which three discrete scenarios are managed at the same time. They introduced a View Correlation Discovery Network (VCDN) to concatenate multi-view data. Liu et al. introduced dynamic pose images (DPI) and attention-based dynamic texture images (att-DTIs) in [45] to obtain spatial and temporal cues. They combined DPI and att-DTIs through multi-stream deep neural networks and a late fusion scheme. Inertial sensor-based low-level and high-level features are used in [46] to categorize human actions acted by a performer in real time. Haider et al. [47] introduced balanced, imbalanced, and super-bagging methods to recognize volleyball action. They used four wearable sensors to evaluate their method. Using signals created by the inertial measurement unit [48] introduced a method based on 1D-CNN construction and consider the tractability of features in time and duration. Bai et al. [49], presented a Collaborative Attention Mechanism (CAM) to develop Multi-view action recognition (MVAR) performance. They also proposed Mutual-Aid RNN (MAR) cell to obtain multi-view sequential information. Ullah et al. [50] introduced a conflux long short-term memory (LSTMs) network. They used CNN model to extract features and used SoftMax for classification. A fusion technique called View-Correlation Adaptation (VCA) in feature and label space was presented in [51]. They generated a semi-supervised feature augmentation (SeMix) and introduced a label-level fusion network. In [52], a light-weight CNN model was used to detect humans and LiteFlowNet CNN was proposed to extract features. The deep skip connection gated recurrent unit (DS-GRU) was used to recognize the action.

## 3. Proposed Recognition Framework

In this segment, we introduced the proposed framework with a detailed discussion on the construction of DMMs sequences, 3D auto-correlation features extraction, and action recognition. Algorithms 1 and 2 describe the mechanism of feature extraction and action recognition, respectively.
**Algorithm 1** Algorithm for feature vector construction**Input:** A Depth action video *D* of frame length *L***Steps:****1.** Split *D* and construct a set ${\left\{S_{j}\right\}}_{j=1}^{m}$, where $len\left(S_{j}\right)=l_{1}$ for all *j***2.** For all sub-sequences $S_{j}$, calculate $DMM_{f}^{j}$, $DMM_{s}^{j}$ and $DMM_{t}^{j}$ through Equation (1)**3.** Use outcomes of **Step2** and generate ${\left\{DMM_{f}^{j}\right\}}_{j=1}^{m}$, ${\left\{DMM_{s}^{j}\right\}}_{j=1}^{m}$ and ${\left\{DMM_{t}^{j}\right\}}_{j=1}^{m}$**4.** Use outcomes of **Step3** and calculate three feature vectors through Equations (3) and (4)**5.** Concatenate outcomes of **Step4****6.** Further split *D* and construct another set ${\left\{V_{k}\right\}}_{k=1}^{n}$, where $len\left(V_{k}\right)=l_{2}$ for all *k***7.** Follow **Step2–Step5** for ${\left\{V_{k}\right\}}_{k=1}^{n}$**Output:** Two auto-correlation feature vectors $H_{1}$ and $H_{2}$

**Algorithm 2** Algorithm for action recognition
**Input:** The training feature set $Y={\left\{y_{j}\right\}}_{j=1}^{n}$, test sample *c*, λ, *K* (number of action classes), class label $k_{i}$ (for class partitioning), *Q* is the number of classifiers.

**Steps:**

**1.** Calculate ${\hat{\gamma }}_{i}$ using Equation (8)

**2. for** Q∈{l,2}

  **for** $c\in \{H_{1},H_{2}\}$← two feature vectors are calculated for *c* using Algorithm 1
   **for all** *i* **do**
    Partition $Y_{i}$,${\hat{\gamma }}_{i}$
    Calculate $e_{i}={∥c-Y_{i}{\hat{\gamma }}_{i}∥}_{2}$
    Calculate $p_{q}\left(\omega |c\right)$ through Equation (11)
   **end for**
  **end for**
 **end for**
   **3.** Calculate P(ω|c) through Equation (12)
   **4.** Decide class(c) through Equation (13)

**Output:** class(c)



### 3.1. Construction of DMMs Sequences

In our work, DMMs corresponding to three projection views (front, side, and top) are constructed for each sub-sequence of depth map sequence. To obtain DMMs, all the depth frames of each sub-sequence are projected onto 3D Euclidean space and projection frames corresponding to three projected views are generated. For each projected view, the addition of the utmost differences between sequential projection frames forms DMMs of front, side, and top.

To interpret computation of DMMs sequence [23], at first, a depth video *D* of length *L* is divided into a set ${\left\{S_{j}\right\}}_{j=1}^{m}$ of sub-sequences of uniform size $l_{1}>0$ as $D=\cup_{j=1}^{m}S_{j}$, where *j* represents the index of sub-sequence. Let us consider a depth frame sequence $\left\{p^{1},p^{2},p^{3},\ldots ,p^{l_{1}}\right\}$ for each sub-sequence, where $l_{1}$ is the frame length of each sub-sequence, i.e., $len\left(S_{j}\right)=l_{1}$ for all *j*. The projection of *i*th frame $p^{i}$ on 3D Euclidean space provides three projected frames $p_{v}^{i}$ (which are referred to as $PF_{v}^{j}$ in Figure 1), where *v* designates front, side, and top projection views and v∈{f,s,t}. The DMMs corresponding to projection views are defined by the following equation:

(1) $$DMM_{v}=\sum_{i=1}^{l_{1}-1}|p_{v}^{i+1}-p_{v}^{i}|,$$

**Figure 2:**
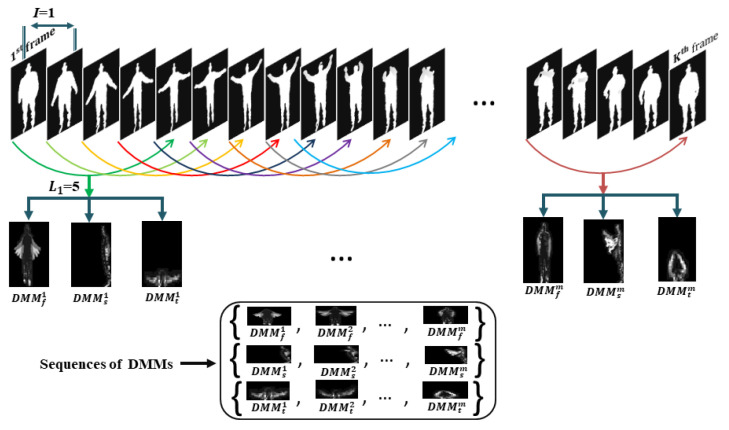
Construction of DMMs sequences according to sub-sequences of 5 frames.

For all $S_{j}$, DMMs are represented by $DMM_{f}^{j}$, $DMM_{s}^{j}$, and $DMM_{t}^{j}$. Therefore, $\{DMM_{f}^{1},$$DMM_{f}^{2},\ldots ,DMM_{f}^{m}\}$, $\left\{DMM_{s}^{1},DMM_{s}^{2},\ldots ,DMM_{s}^{m}\right\}$ and $\left\{DMM_{t}^{1},DMM_{t}^{2},\ldots ,DMM_{t}^{m}\right\}$ sequences are formed from ${\left\{S_{j}\right\}}_{j=1}^{m}$ of *D*. In action datasets, the same actions are performed by different individuals with different speeds. To cope with action speed variations, the depth map sequence of *D* is further divided into another set ${\left\{V_{k}\right\}}_{k=1}^{n}$ of sub-sequences where frame length of each sub-sequence is $l_{2}>0$, i.e., $len\left(V_{k}\right)=l_{2}$ for all *k* (see Figure 1). As a result, more three new sets of DMMs sequences $\left\{DMM_{f}^{1},DMM_{f}^{2},\ldots ,DMM_{f}^{n}\right\}$, $\left\{DMM_{s}^{1},DMM_{s}^{2},\ldots ,DMM_{s}^{n}\right\}$ and $\left\{DMM_{t}^{1},DMM_{t}^{2},\ldots ,DMM_{t}^{n}\right\}$ are obtained from ${\left\{V_{k}\right\}}_{k=1}^{n}$. In our DMMs sequences constructing mechanism, numerical values of frame lengths $l_{1}$ and $l_{2}$ are experimentally chosen to 5 and 10 respectively. The frame length of a sub-sequence may vary and must be set to less than the length of total depth video, i.e., $l_{1}$ and $l_{2}<L$. The DMMS sequences constructing scheme for frames length $l_{1}$ is displayed in Figure 2. The frame interval *I* in Figure 2 is set to 1 which is the number of frames from the first frame of a portion to the first frame of the neighboring portion which indicates how many frames between the two portions are overlapped. Please note that the frame interval must be less than the frame length of a sub-sequence, i.e., $I<l_{1}$ and $l_{2}$.

### 3.2. Action Vector Formation

**Figure 3:**
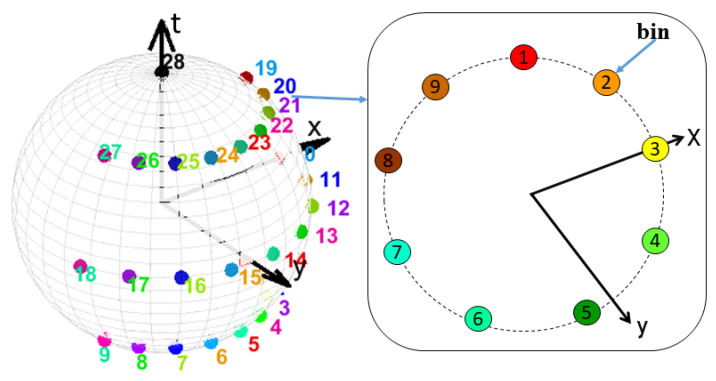
28 orientation bins along latitude and longitude on a hemisphere. 4 orientation bin layers including a layer at pole are in two-dimensional x−y plane. 9 orientation bins are located on each layer except at the pole (contains one bin).

STACOG was introduced in [53] for RGB video sequences to extract local relationships within the space-time gradients of three-dimensional motion by using auto-correlation functions to space-time orientations and the magnitudes of the gradients. In our work, this method is applied to all the DMMs sequences (calculated in the previous section) of a depth video *D* to extract 3D geometric features of human motion. At each space-time volume S(x,y,t) (in general, this volume stands for a DMMs sequence) around each space-time point in a DMMs sequence the space-time gradient vector is computed through the derivatives $S_{x}$, $S_{y}$, and $S_{t}$ to extract features. The space-time gradients can be described by angles α = arctan($S_{x},S_{y}$) and β = arcsin($\frac{S_{t}}{m_{g}}$), where the magnitude of gradient is defined by $m_{g}=\sqrt{(}S_{x}^{2}+S_{y}^{2}+S_{t}^{2})$. By the two angles, space-time orientation of the gradient is coded into *B* orientation bins on a unit sphere by selecting weights to the nearest bins (see Figure 3). Finally, the orientation is represented by *B*-dimensional vector named space-time orientation coding (STOC) vector which is denoted by b. By using the magnitude $m_{g}$ and the STOC vector b of the gradients, the *N*th order auto-correlation function for the space-time gradients is defined as follows:

(2) $$\begin{array}{c} R_{N}\left(d_{1},\ldots ,d_{N}\right)=\int f\left[m_{g}\left(p\right),\ldots ,m_{g}\left(p+d_{N}\right)\right]b\left(p\right)⊗\cdots ⊗b\left(p+d_{N}\right)dp \end{array}$$

where $d_{i}=\left(d_{1},\ldots ,d_{N}\right)$ displacement are vectors from the reference point p=(x,y,t), *f* represents a weighting function and ⊗ is the tensor product of vector. In the tensor products, there are small numbers of non-zero components related to the gradient orientations of the neighboring vectors. The parameters $N\in \left\{0,1\right\};d_{1x,y}\in \left\{\pm \Delta s,0\right\};d_{1t}\in \left\{\Delta t,0\right\};f\left(\cdot \right)\equiv \min\left(\cdot \right)$ are confined in the experiment. Where Δs is the displacement interval along the spatial axis and Δt is that of along the temporal axis. To inhibit the effect of isolated noise on surrounding auto-correlations, min is received regarding to weight function *f*.

For N∈{0,1} the 0th order and the 1st order STACOG features can be written as,

(3) $$S_{0}=\sum_{p}m_{g}\left(p\right)b\left(p\right),$$

(4) $$S_{1}\left(d_{1}\right)=\sum_{p}\min\left[m_{g}\left(p\right),m_{g}\left(p+d_{1}\right)\right]b\left(p\right)b{\left(p+d_{1}\right)}^{T},$$

where $S_{0}$ and $S_{1}$ are 0th and 1st order auto-correlations which gives the 0th order and the 1st order STACOG features, and *T* is the transpose.

### 3.3. Action Recognition

By applying Algorithm 1, two auto-correlation feature vectors $H_{1}$ and $H_{2}$ are acquired corresponding to two different sets of sub-sequences, ${\left\{S_{j}\right\}}_{j=1}^{m}$ and ${\left\{V_{k}\right\}}_{k=1}^{n}$, of the depth video *D* (see Figure 1). The dimension of $H_{1}$ and $H_{2}$ are reduced through Principal Component Analysis (PCA) [54]. Then the two vectors are passed separately to $L_{2}$-regularized Collaborative Representation Classifier ($L_{2}$-CRC) [13] and the relevant two distinct outcomes are fused by logarithmic opinion pool (LOGP) [14]. To explain $L_{2}$-CRC, let us denote the class number by *K*. The set $Y=\left[Y_{1},Y_{2},Y_{3},\ldots ,Y_{i},\ldots ,Y_{K}\right]=\left[y_{1},y_{2},y_{3},\ldots ,y_{j},\ldots ,y_{n}\right]\in R^{\left(d\times n\right)}$ is the set of all training samples, where *d* is the-dimensionality of training samples, *m* is the number of training samples from *K* classes, $Y_{i}\in R^{\left(d\times m_{i}\right)}$ is subset of training samples from class *i* and $y_{j}\in R^{d}$ is any training sample of $Y_{i}$. Let, $c\in R^{d}$ be any unknown test sample which is defined by the linear combination of all the training samples in *Y*:

(5) c≈Yγ,

where $\gamma =[\gamma_{1},\gamma_{2},\gamma_{3},\cdots ,\gamma_{i},\ldots ,\gamma_{K}]$ is a m×1 coefficients vector associated with the training samples of class *i*. In practice, Equation(5) cannot be solved directly because it is under determination [55]. By the solution of the following norm minimization problem, Equation(5) can be solved:

(6) $$\begin{array}{cc} \arg\;\min_{\gamma }{\{∥c-Y\gamma ∥}_{2}^{2}+\lambda ∥M\gamma {∥}_{2}^{2}\},\\ \mathrm{subject}\mathrm{to}\; & c\approx Y\gamma , \end{array}$$

where λ denotes the regularization parameter and *M* is the Tikhonov regularization matrix [56], which is configured by the following diagonal matrix.

(7) $$M=\left[\begin{array}{ccc} {\left|\left|c-y_{1}\right|\right|}_{2} & \cdots & 0\\ \vdots & \ddots & \vdots \\ 0 & \cdots & {\left|\left|c-y_{n}\right|\right|}_{2} \end{array}\right],$$

The coefficient vector can be calculated as [57],

(8) $$\hat{\gamma }={\left(Y^{T}Y+\lambda M^{T}M\right)}^{-1}Y^{T}c=Zc,$$

Since the training samples are *Y* is given and λ is determined by these samples then *Z* can be simply calculated and thus *Z* is independent of *c*. It is clear when the test sample *c* is given, the corresponding vector $\hat{\gamma }$ can be easily computed from Equation (8). The coefficient vector $\hat{\gamma }$ is represented as $\left[{\hat{\gamma }}_{1},{\hat{\gamma }}_{2},{\hat{\gamma }}_{3},\ldots ,{\hat{\gamma }}_{i},\ldots ,{\hat{\gamma }}_{K}\right]$ by considering all the action classes. Now, the class-specific residual error can be obtained by

(9) $$e_{i}={∥c-Y_{i}{\hat{\gamma }}_{i}∥}_{2},$$

where, $Y_{i}$ is the dictionary sample and ${\hat{\gamma }}_{i}$ is the coefficient of *i*th class, respectively.

From Equation (9), an error vector is obtained about an input feature vector. In our case, there are two error vectors $e^{1}=\left[e_{1}^{1},e_{2}^{1},e_{3}^{1},\ldots ,e_{i}^{1},\ldots ,e_{K}^{1}\right]$ and $e^{2}=\left[e_{1}^{2},e_{2}^{2},e_{3}^{2},\ldots ,e_{i}^{2},\ldots ,e_{K}^{2}\right]$ since we input two feature vectors $H_{1}$ and $H_{2}$ obtained by Algorithm 1 for the test sample *c*. A decision fusion scheme logarithmic opinion pool (LOGP) [14] rule is used to concatenate the probabilities of those errors and to output the class label. In this scheme, the following global membership function is calculated through the posterior probability $p_{q}\left(\omega |c\right)$ of each classifier.

(10) $$P\left(\omega |c\right)=\prod_{q=1}^{Q}p_{q}{\left(\omega |c\right)}^{\frac{1}{Q}},$$

where ω∈[1,2,3,…,i,…,K] denotes class label, and Q(=2) denotes the number of classifiers.

Then a Gaussian mass function corresponding to the residual error $e=[e_{1},e_{2},e_{3},\ldots ,e_{i},\ldots ,e_{K}]$ is represented by the following equation.

(11) $$p_{q}\left(\omega |c\right)\approx \exp\left(-e_{i}\right),$$

Equation (11) defines the higher posterior probability $p_{q}\left(\omega |c\right)$ for a smaller residual error $e_{i}$. Therefore, the combined probability from the two classifiers is defined as:

(12) $$P\left(\omega |c\right)=\exp{\left(-e_{i}^{1}\right)}^{\frac{1}{2}}\times \exp{\left(-e_{i}^{2}\right)}^{\frac{1}{2}},$$

(13) $$\mathrm{And},\;class\left(c\right)=\max\left\{P(\omega |c)\right\},$$

where $e^{1}$ and $e^{2}$ are normalized to [0,1].

## 4. Experimental Results and Discussion

This section discusses three sets of experiments on three datasets to evaluate the performance of the proposed framework. First, the datasets are introduced along with their challenges. Secondly, the setup of STACOG parameters is then discussed to evaluate the proposed framework. Finally, experimental results on three datasets are described.

### 4.1. Datasets

Our proposed framework is greatly appraised on depth-based actions datasets named MSR-action 3D dataset [58], DHA dataset [59], and UTD-MHAD dataset [38].

#### 4.1.1. MSR-Action 3D Dataset

MSR-Action 3D dataset is captured by a depth camera which represents action data of depth map sequences. The resolution of each map is 320×240. This dataset has 20 types of action categories. All the actions are acted by 10 different persons and every subject act in each action 2 or 3 times. In this dataset, the number of depth map sequences is 557 [58]. This dataset is a challenging because of the correspondence between some actions (e.g., *“Draw x”* and *“Draw tick”*).

#### 4.1.2. DHA Dataset

DHA dataset was introduced in [59] which contains some actions extended from the Weizmann dataset [60]. The Weizmann dataset is used in action recognition based on RGB sequences. The DHA dataset involves 23 action types among which 1 to 10 actions are adopted from Weizmann dataset [61]. All the actions are performed by 21 subjects (12 males and nine females) and the total number of depth map sequences is 483. Because of the inter-similarity between action classes (e.g., *“rod-swing”* and *“golf swing”*), the DHA dataset is challenging.

#### 4.1.3. UTD-MHAD Dataset

In the UTD-MHAD dataset [38], RGB videos, depth videos, skeleton positions, and inertial signals are captured by a video sensor and a wearable inertial sensor. All the actions of this dataset contain 27 actions and all the actions are performed by eight subjects (four females and four males). Each performer repeats each action four times. This dataset includes 861 depth action sequences, after eliminating three inappropriate sequences.

### 4.2. Parameter Setting

The proposed framework is evaluated on the datasets discussed above and compared with the other state-of-the-art approaches. Of all the samples of each dataset, some samples are used as training samples and the remaining samples are used as test samples. Depending on the test samples, results on all datasets are obtained. Each depth action video of all datasets is partitioned into sub-sequences using the same frame lengths. The frame interval between two consecutive sub-sequences is set to 1 which indicates the number of overlapping frames. Thus, for two different frame lengths 5 and 10, the overlapping frames 4 and 9 are obtained, respectively. Additionally, for all action datasets, we used the same values of parameters. At first, all parameter values are tuned for a dataset to query which values give the highest recognition accuracy. Then, the values of parameters set for the highest result are used in all other datasets to verify the superiority of the framework. To extract STACOG features, orientation bins in the x−y plane and orientation bin layers are set to 9 and 4, respectively. According to [15], the temporal interval is set to 1 and the spatial interval is fixed to 8. The $L_{2}$-CRC parameter λ is tuned to 0.0001.

### 4.3. Classification on MSR-Action 3D Dataset

**Figure 4:**
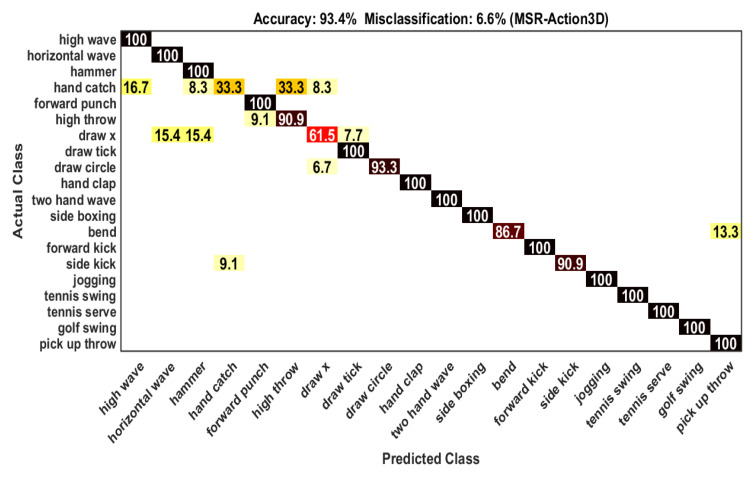
Confusion matrix on MSR-Action 3D dataset.

In the experimental arrangement, we used all action categories of MSR-Action 3D dataset instead of dividing them into different action subsets. The action samples acted through persons of the odd number are employed as training samples (284) and the samples of the remaining persons of even number are used as test samples(273). Our proposed framework gives 93.4% recognition accuracy which is compared with other frameworks on depth data as shown in Table 1. Among 20 actions, the classification accuracy is 100% for 14 actions. The remaining 6 actions have some confusion with other actions because of some inter-class similarities. For example, the confusion of an action *“Side kick”* with an action *“Hand catch”* is 9.1% (see Figure 4). The accuracy including confusion information of each class is further clarified in Table 2.

### 4.4. Classification on DHA Dataset

**Figure 5:**
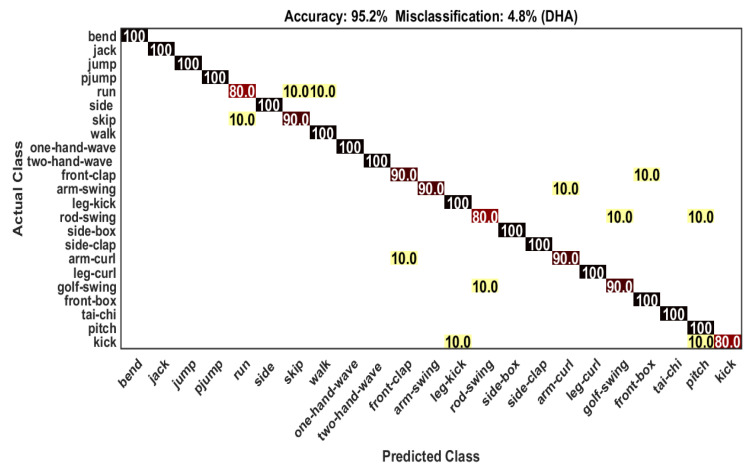
Confusion matrix on DHA dataset.

In the DHA dataset, samples of the odd subjects are used as training samples and the samples of the even subjects are used as test samples. There are 253 samples are used as training samples and 230 samples are used as test samples. Our proposed framework achieves 95.2% accuracy which shows the effectiveness of the recognition framework. From Table 3, we can observe that 15 out of 23 actions are recognized with 100% accuracy. The remaining 8 actions are confused with other actions shown in Figure 5. The action *“golf swing”* gives 10% confusion with *“rod-swing”*. The comparison of our recognition framework with other state-of-the-art methods is shown in Table 3. It is clear from the table that our proposed framework beats other existing frameworks considerably. The class-wise classification accuracy (for right and wrong classification) is shown in Table 4.

### 4.5. Classification on UTD-MHAD Dataset

**Figure 6:**
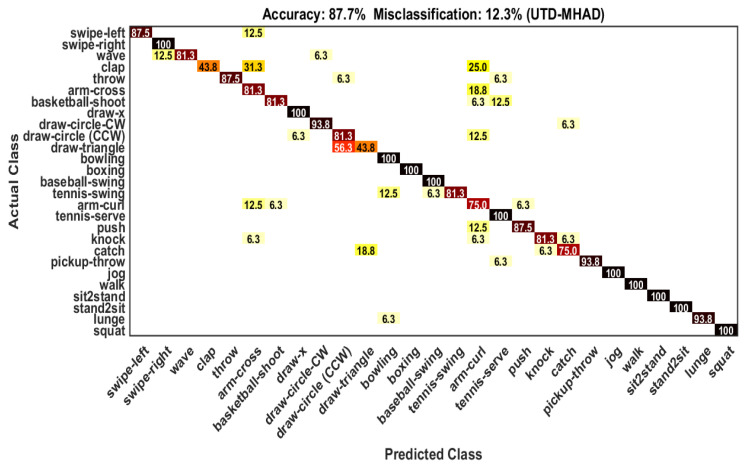
Confusion matrix on the UTD-MHAD dataset.

In the UTD-MHAD dataset, samples of the odd subjects are used as training samples (431) and the samples of the even subjects are used as test samples (430). The evaluation result of our framework on this dataset gives 87.7% recognition accuracy (see Table 5) because of using varieties actions. The result in our recognition framework gives 100% accuracy for 11 actions and the remaining 16 actions show confusion with other actions (see Figure 6). The individual class recognition performance is reported in Table 6.

### 4.6. Efficiency Evaluation

The execution time and the space complexity of key factors are deliberated to show the efficiency of our system.

#### 4.6.1. Execution Time

The system is executed by using MATLAB on CPU platform with an Intel i5-7500 Quad-core processor of 3.41 GHz frequency and a RAM of 16 GB. There are seven major components in the proposed approach: DMMs sequences construction for frame length 5, DMMs sequences construction for frame length 10, $H_{1}$ feature vector generation, $H_{2}$ feature vector generation, PCA on $H_{1}$, PCA on $H_{2}$, Action label. The execution time of these components is determined to assess the time efficiency of the system on three datasets as MSR-Action 3D, DHA, and UTD-MHAD dataset. Table 7 showed the execution time (in milliseconds) of the seven components on those datasets and compared the total execution time on the datasets. In the case of the MSR-Action 3D dataset, execution times are calculated for each action sample with 40 frames on average. As can be seen from Table 7, 40 frames are processed in less than one second (i.e., 252.6 ± 74.8 milliseconds). Therefore, our proposed recognition framework can be used for real-time action recognition on the MSR-Action 3D dataset. The execution times on the DHA dataset are calculated for each action sample with 29 frames on average. Table 7 showed that the 29 frames are processed in less than one second (i.e., 379.1 ± 90.7 milliseconds) which proves our framework can be used for real-time action recognition on the DHA dataset. Table 7 also presented the execution times (in milliseconds) on the UTD-MHAD dataset for each action sample with 68 frames on average. To process 68 frames, the system requires less than one second (i.e., 508.9 ± 100.9 milliseconds) which showed the capability of the real-time action recognition of our proposed framework.

#### 4.6.2. Space Complexity

The components PCA and $L_{2}$-CRC are the key components for the calculation of space complexity of the proposed system. PCA and $L_{2}$-CRC are adopted for both frame lengths 5 and 10. Therefore, the complexity of PCA is $2*O\left(l^{3}+l^{2}m\right)$ [23] and the complexity of $L_{2}$-CRC is $2*O(n_{c}\times m)$ [62]. Thus, the total complexity of the system can be expressed as $2*O\left(l^{3}+l^{2}m\right)+\;2*O\left(n_{c}\times m\right)$. Table 8 describes the computed complexity and compared it with the complexities of other existing frameworks. The table shows that our framework delivers lower complexity and recognizes actions better than other existing frameworks.

## 5. Conclusions

In this paper, we present an effective action recognition framework that is based on 3D Auto-Correlation features. In fact, the Depth Motion Maps (DMMs) sequence representation is firstly introduced to obtain additional temporal motion information from depth map sequences which can distinguish similar actions. The space-time auto-correlation of gradients features description algorithm is then used to extract motion cues from the sequences of DMMs according to different projection views. At last, the Collaborative representation classifier (CRC) and the decision fusion scheme are used for detecting action class. Experimental results on three benchmark datasets shows that the proposed framework is better than the state-of-the-art methods. Moreover, the framework outperforms other existing techniques that are based on space-time auto-correlation of gradients feature. Furthermore, the space-time complexity analysis of the proposed framework indicates that it can be used for the real-time human action recognition.

## Figures and Tables

**Table 1:** Comparison of action recognition accuracy (%) with state-of-the-art frameworks on the MSR-Action 3D dataset.

Approach	Accuracy (%)
Decision-level Fusion (MV) [19]	91.9
DMM-GLAC-FF [16]	89.38
DMM-GLAC-DF [16]	92.31
DMM-LBP-FF [21]	91.9
DMM-LBP-DF [21]	93.0
$3D^{2}$CNN [41]	84.07
Skeleton-MSH [39]	90.98
3DHoT_S [28]	91.9
3DHoT_M [28]	88.3
Depth-STACOG [15]	75.82
DMM-GLAC [15]	89.38
WDMM [32]	90.0
DMM-UDTCWT [35]	92.67
Proposed Approach	**93.4**

**Table 2:** Class-specific accuracy on MSR-Action3D dataset.

Actions	Classification (%)	Confusion (%)
High wave	100	No confusion
Horizontal wave	91.7	Hammer (8.3)
Hammer	100	No confusion
Hand catch	33.3	High wave (16.7), Hammer (8.3), High throw (33.3), Draw x (8.3)
Forward punch	100	No confusion
High throw	90.9	Forward punch (9.1)
Draw x	61.5	Horizontal wave (15.4), Hammer (15.4), Draw tick (7.7)
Draw tick	100	No confusion
Draw circle	93.3	Draw X (6.7)
Hand clap	100	No confusion
Two hand wave	100	No confusion
Side boxing	100	No confusion
Bend	86.7	Pick up and throw (13.3)
Forward kick	100	No confusion
Side kick	90.9	Hand catch (9.1)
Jogging	100	No confusion
Tennis swing	100	No confusion
Tennis serve	100	No confusion
Golf swing	100	No confusion
Pick up and throw	100	No confusion

**Table 3:** Comparison of action recognition accuracy (%) with state-of-the-art frameworks on the DHA dataset.

Approach	Accuracy (%)
SDM-BSM [20]	89.50
GTI-BoVW [24]	91.92
Depth WDMM [32]	81.05
RGB-VCDN [44]	84.32
VCDN [44]	88.72
Binary Silhouette [43]	91.97
DMM-UDTCWT [35]	94.2
Stridden DMM-UDTCWT [35]	94.6
VCA [51]	89.31
CAM [49]	87.24
Proposed Approach	**95.2**

**Table 4:** Class-specific accuracy on DHA dataset.

Actions	Classification (%)	Confusion (%)
Bend	100	No confusion
Jack	100	No confusion
Jump	100	No confusion
Pjump	100	No confusion
Run	80.0	Skip (10.0), Walk (10.0)
Side	100	No confusion
Skip	90.0	Run (10.0)
Walk	100	No confusion
One-hand-wave	100	No confusion
Two-hand-wave	100	No confusion
Front-clap	90.0	Front-box (10.0)
Arm-swing	90.0	Arm-curl (10.0)
Leg-kick	100	No confusion
Rod-swing	80.0	Golf-swing (10.0), Pitch (10.0)
Side-box	100	No confusion
Side-clap	90.0	Side-box (10.0)
Arm-curl	90.0	Front-clap (10.0)
Leg-curl	100	No confusion
Golf-swing	90.0	Rod-swing (10.0)
Front-box	100	No confusion
Tai-chi	100	No confusion
Pitch	100	No confusion
Kick	90.0	Pitch (10.0)

**Table 5:** Comparison of action recognition accuracy (%) with state-of-the-art frameworks on the UTD-MHAD dataset.

Approach	Accuracy (%)
Kinect [38]	66.10
Inertial [38]	67.20
Kinect+Inertial [38]	79.10
DMM-EOH [19]	75.3
DMM-LBP [19]	84.20
CNN-Top [40]	74.65
CNN-Fusion [40]	86.97
3DHOT-MBC [28]	84.40
VDDMMs [27]	85.10
Structured body DDI [42]	66.05
Structured part DDI [42]	78.70
RGB DTIs [45]	85.39
Inertial [48]	85.35
Proposed Approach	**87.7**

**Table 6:** Class-specific accuracy on UTD-MHAD dataset.

Actions	Classification (%)	Confusion (%)
Swipe-lift	87.5	Arm-cross (12.5)
Swipe-right	100	No confusion
Wave	81.3	Swipe-right (12.5), Draw-circle-CW (6.3)
Clap	43.8	Arm-cross (31.3), Arm-curl (25.0)
Throw	87.5	Draw-circle (CCW) (6.3), Tennis-serve (6.3)
Arm-cross	81.3	Arm-curl (18.8)
Basketball-shoot	81.3	Arm-curl (6.3), Tennis-serve (12.5)
Draw-x	100	No confusion
Draw-circle CW	93.8	Catch (6.3)
Draw-circle (CCW)	81.3	Draw X (6.3), Arm-curl (15.5)
Draw-triangle	43.8	Draw-circle (CCW) (56.3)
Bowling	100	No confusion
Boxing	100	No confusion
Baseball-swing	100	No confusion
Tennis-swing	81.3	Bowling (12.5), Baseball-swing (6.3)
Arm-curl	75.0	Arm-cross (12.5), Basketball-shoot (6.3), Push (6.3)
Tennis-serve	100	No confusion
Push	87.5	Arm-curl (12.5)
Knock	81.3	Arm-cross (6.3), Arm-curl (6.3), Catch (6.3)
Catch	75.0	Draw-triangle (18.8), Knock (6.3)
Pickup-throw	93.8	Tennis-serve (6.3)
Jog	100	No confusion
Walk	100	No confusion
Sit2stand	100	No confusion
Stand2sit	100	No confusion
Lunge	93.8	Bowling (6.3)
Squat	100	No confusion

**Table 7:** Comparison of execution time (mean ± std) of the key factors on three datasets.

Main Components	MSR-Action3D Dataset	DHA Dataset	UTD-MHAD Dataset
DMMs sequences construction for frame length 5	11.3 ± 0.7	80.7 ± 6.2	36.6 ± 3.0
DMMs sequences construction for frame length 10	18.8 ± 1.3	155.6±12.0	67.9 ± 5.5
$H_{1}$ feature vector generation	108.5 ± 35.0	69.9 ± 35.0	197.7 ± 45.6
$H_{2}$ feature vector generation	95.6 ± 36.7	57.2 ± 36.7	180.9 ± 45.9
PCA on $H_{1}$	8.5 ± 0.4	7.3 ± 0.3	10.7 ± 0.3
PCA on $H_{2}$	8.4 ± 0.3	7.2 ± 0.3	10.7 ± 0.3
Action label	1.5 ± 0.4	1.2 ± 0.2	4.4 ± 0.3
**Total execution time**	252.6 ± 74.8/action sample (40 frames)	379.1 ± 90.7/action sample (29 frames)	508.9 ± 100.9/action sample (68 frames)

**Table 8:** Comparison of computational complexity of the proposed approach with other existing approaches.

Approach	Components	Space Complexity
DMM [23]	PCA, $L{}_{2}$-CRC	$O\left(l^{3}+l^{2}m\right)+O\left(n_{c}\times m\right)$ *l* = size of action vector, *m* = number of training samples, $n_{c}$ = number of action classes
DMM-LBP-DF [21]	PCA, Kernel-based Extreme Learning Machine (KELM)	$O\left(l^{3}+l^{2}m\right)+3*O\left(m^{3}\right)$ *l* = size of action vector, *m* = number of training samples
MHF+SHF+KELM [13]	PCA, KELM	$O\left(l^{3}+l^{2}m\right)+2*O\left(m^{3}\right)$ *l* = size of action vector, *m* = number of training samples
GMSHI+GSHI+CRC [36]	PCA, $L{}_{2}$-CRC	$O\left(l^{3}+l^{2}m\right)+\;O\left(n_{c}\times m\right)$ *l* = size of action vector, *t* = number of training samples, $n_{c}$ = number of action classes
Enhanced auto-correlation [63]	PCA, KELM ensemble	$O\left(l^{3}+l^{2}m\right)+O\left(m^{3}\right)$ *l* = size of action vector, *m* = number of action classes
**Proposed Approach**	PCA, $L{}_{2}$-CRC	$2*O\left(l^{3}+l^{2}m\right)+\;2*O\left(n_{c}\times m\right)$ *l* = size of action vector, *m* = number of training samples, $n_{c}$ = number of action classes
